# Comparison of Fecal Microbiota and Metabolites Between Captive and Grazing Male Reindeer

**DOI:** 10.3390/ani14243606

**Published:** 2024-12-14

**Authors:** Fei Zhao, Quanmin Zhao, Songze Li, Yuhang Zhu, Huazhe Si, Jiang Feng, Zhipeng Li

**Affiliations:** 1 College of Life Science, Jilin Agricultural University, Changchun 130118, China; zq88467433@163.com; 2 College of Animal Science and Technology, Jilin Agricultural University, Changchun 130118, China; ze961672411@163.com (S.L.); yuhangzhu199512@163.com (Y.Z.); sihuazhe1989@163.com (H.S.); 3 College of Chinese Medicinal Materials, Jilin Agricultural University, Changchun 130118, China; qm3657@163.com; 4 Jilin Provincial Key Laboratory of Animal Resource Conservation and Utilization, Northeast Normal University, Changchun 130117, China; 5 Key Lab of Animal Production, Product Quality and Security, Ministry of Education, Jilin Agricultural University, Changchun 130118, China

**Keywords:** reindeer, full-length 16S rRNA gene, metabolome, summer, captivity

## Abstract

The reindeer (*Rangifer tarandus*) is a circumpolar member of the Cervidae family, and has adapted to a harsh environment. Summer is a critical period for reindeer, with peak digestibility facilitating body fat accumulation. The gut microbiota plays a pivotal role in nutrient metabolism, and is affected by captivity. However, differences in the composition of the gut microbiota and metabolites between captive and grazing reindeer during summer remain poorly understood. Here, we conducted a comparative study of the fecal microbiota and metabolites between captive (*n* = 6) and grazing (*n* = 6) male reindeer, using full-length 16S rRNA gene sequencing and gas chromatography–time-of-flight mass spectrometry, respectively. Our results indicated that *Prevotella*, *Phocaeicola*, *Papillibacter*, *Muribaculum*, and *Bacteroides* were the predominant genera in the feces of reindeer. However, microbial diversity was significantly higher in captive reindeer compared to their grazing counterparts. Principal coordinate analysis revealed significant differences in the fecal microbiota between captive and grazing reindeer. In captive reindeer, the relative abundances of the genera *Clostridium*, *Paraprevotella*, *Alistipes*, *Paludibacter*, *Lentimicrobium*, *Paraclostridium*, and *Anaerovibrio* were significantly higher, while those of the genera *Prevotella*, *Phocaeicola*, *Pseudoflavonifractor*, and *Lactonifactor* were significantly lower. A comparison of predicted functions indicated that pathways involved in fat digestion and absorption, histidine metabolism, lysine biosynthesis, and secondary bile acid biosynthesis were more abundant in captive reindeer, whereas the pathways of fructose and mannose metabolism and propanoate metabolism were less abundant. An untargeted metabolomic analysis revealed that 624 metabolites (e.g., amino acids, lipids, fatty acids, and bile acids) and 645 metabolites (e.g., carbohydrates and purines) were significantly increased in the feces of captive and grazing reindeer, respectively. In conclusion, we unveiled significant differences in fecal microbiota and metabolites between captive and grazing male reindeer, with the results suggesting a potentially enhanced ability to utilize plant fibers in grazing reindeer.

## Simple Summary

Reindeer are primarily distributed across circumpolar regions, and must receive adequate nutrition in order to accumulate body fat during the summer. The gut microbiota plays a vital role in nutrient metabolism. However, differences in the gut microbial composition and metabolic profile between captive and grazing male reindeer during summer need to be further investigated. Our results demonstrated that the gut microbial communities, functions, and metabolic profiles significantly differed between captive and grazing reindeer. Fecal microbes responsible for the metabolism of amino acids and fatty acids were enriched in captive reindeer, while those associated with fiber utilization were abundant in grazing reindeer. These findings suggest that captivity leads to alterations in the gut microbiota and metabolites in reindeer.

## 1. Introduction

The reindeer (*Rangifer tarandus*), a unique member within the Cervidae family, is widely distributed across the boreal, tundra, subarctic, arctic, and alpine regions of northern Asia, North America, and Europe [1]. Reindeer have played a crucial role in human civilizations by providing food and clothing to Eurasian Arctic and Subarctic communities [2]. However, reindeer face extreme temperatures and limited food availability during winter, resulting in starvation and death [3]. To adapt to these harsh conditions, reindeer lower their metabolic rate and rely on body fat and, in extreme cases, muscle protein for energy production [4]. Thus, reindeer must accumulate sufficient energy during the summer, which is when their feed intake and digestibility reach their peaks [5], resulting in a significant increase in body weight and body fat [6]. Meanwhile, the concentrations of glucose, triglycerides, total lipids, and total protein in the blood also increase during summer relative to winter [7]. Additionally, enzyme activities linked to fatty acid β-oxidation and lactate metabolism in the muscle significantly increase [8]. Hence, it is important to understand the nutrient metabolism in the gut of reindeer during summer.

A growing body of evidence demonstrates that the gut microbiota plays a pivotal role in nutrient metabolism and physiology [9]. Culture methods have revealed that, in the ceca of reindeer, cellulose-degrading bacteria, including *Butyrivibrio fibrisolvens*, *Ruminococcus albus*, and *Prevotella ruminicola*, and starch-degrading bacteria, such as *Streptococcus bovis* and *Selenomonas ruminantium*, potentially contribute to the digestion of plant materials in both summer and winter [10]. However, significant changes in certain minor communities have been found in adult reindeer, with an increase in the proportion of the phylum Actinobacteriota in summer and autumn, compared to in winter and spring, suggesting that the rumen microbiota can improve the ability of reindeer to utilize nutrients with low availability [1]. In addition, we have demonstrated geographical differences between the gastrointestinal tract microbiota of different ruminants [11]. Testing fecal samples is non-invasive and causes minimal interference with natural populations of reindeer [12]. Interestingly, evidence suggests that the gut microbiota can be considered an endocrine organ involved in the maintenance of energy homeostasis, affecting fat storage in the host [13]. Moreover, metabolomics is regarded as a key approach to detecting and identifying small molecules produced by the gut microbiota, and to understanding the functions of microbes in the gut [14]. These findings highlight the importance of revealing the fecal microbiota and metabolome of reindeer during the summer.

Captivity also has an important influence on the gut microbial composition, resulting in changes in microbial diversity relative to free-living populations [15]. It has been observed that the feces of woodrats, collected from the live traps used for their capture, exhibited higher microbial diversity than samples collected from the same animals after two weeks in captivity, which is attributed to a loss of microbial diversity in captivity [16]. However, free-ranging red deer exhibited lower microbial diversity compared with red deer in enclosures, probably because of the supplementation of the food available to the animals [17,18]. A remarkable finding was a significant reduction in the phylum Firmicutes, as well as a slightly higher abundance of Bacteroidetes, in the rumen of adult female roe deer with supplemental feeding, in comparison with free-ranging roe deer [19]. Similarly, the feces of captive sika deer also showed higher microbial diversity than wild populations, corresponding to higher Bacteroidetes and lower Firmicutes abundances in captive sika deer [20]. It was reported that captive reindeer gained significant body weight and had greater rumen weights than grazing reindeer [21]. Interestingly, Mönttinen et al. (2022) found that microbial diversity and the relative abundances of Rikenellaceae and Oscillospiraceae were significantly higher in captive reindeer, whereas Bifidobacteriales and Prevotellaceae were markedly less abundant compared to in grazing reindeer during winter [22]. Thus, we hypothesized that the fecal microbiota and metabolites differ between captive and grazing reindeer during summer.

In this study, we aimed to investigate and identify differences in fecal microbiota and metabolites between captive and grazing male reindeer by performing full-length 16S rRNA gene sequencing and ultra-high-performance liquid chromatography, respectively. Our results provide a comprehensive understanding of the fecal microbial communities and metabolic profiles in male reindeer in summer, and demonstrate the impact of captivity on the fecal microbiota and its functional profiles.

## 2. Materials and Methods

### 2.1. Animals and Sample Collection

In this study, we utilized a cohort of 12 healthy, three-year-old male reindeer, comprising 6 captive and 6 grazing reindeer, because age and sex affect the gut microbiota [15]. The captive reindeer, designated as the Cap group, were housed in individual pens (3 m × 3 m) at the reindeer research station in Jilin Agricultural University (43°48′ N, 125°24′ E), with a mean daily temperature varying between 12 °C and 21 °C, and were fed a total mixed ration consisting of alfalfa and concentrate (45:55, dry matter basis, Table S1) twice daily. They also had unrestricted access to clean drinking water. The grazing reindeer, referred to as the Gra group, were located in the Khama National Nature Reserve in Inner Mongolia, China (51°20′ N, 122°23′ E), with a mean daily temperature varying between 1 °C and 16 °C; they primarily subsisted on foraged woody plants, graminoids, mosses, and lichens. The reindeer were followed until fresh feces were produced, and the fecal pellets (>10) from a single defecation event were collected and separately stored in the sterilized tubes. The outer layers of the feces were removed with a sterilized scalpel to minimize environmental contamination [17]. The inner parts were frozen in liquid nitrogen and stored at −80 °C. All animal procedures were reviewed and approved by the Animal Ethics Committee of Jilin Agricultural University (No.20230421001).

### 2.2. DNA Extraction, 16S rRNA Gene Sequencing, and Bioinformatic Analysis

The feces were thawed, and five pellets were randomly selected from each fecal sample and homogenized using the MP FastPrep-24 (MP Biomedicals, Illkrich, France). Total genomic DNA was extracted from 200 mg of feces per sample using the QIAamp^®^ Fast DNA Stool Mini Kit (QIAGEN, Valencia, CA, USA), along with the FastPrep-24 (MP Biomedicals, Illkrich, France). The integrity and quantity of the DNA were confirmed using 1.0% agarose gel electrophoresis and a NanoDrop ND-1000 spectrophotometer (Thermo Scientific, Wilmington, NC, USA). Subsequently, the full-length 16S rRNA gene was amplified with the primers 27F and 1492R, where both the forward and reverse primers were tailed with an 8 bp barcode sequence added to each sample. The resulting amplicons were purified using the AxyPrepDNA Gel Extraction Kit (Axygen Biosciences, Union City, CA, USA), and their concentrations were measured with the QuantiFluor^™^-ST (Promega Corporation, Madison, WI, USA). Amplicon libraries were constructed following the guidelines of the SMRTbell Express TPK 2.0 Kit (Pacific Biosciences, New York, NY, USA). These libraries were then sequenced on the PacBio Sequel II platform. Circular consensus sequences (CCSs) were generated utilizing the SMRT Link (v9.0) Analysis software, with parameters set to a minimum predicted accuracy of 0.99 and a minimum of three passes.

Generated CCS reads with lengths of >1800 bp or <1200 bp were excluded using the SMRT Portal. The remaining sequences were clustered into operational taxonomic units (OTUs) at a 98.65% similarity threshold, using UPARSE implemented in USEARCH (v11.0.667) [23]. Potential chimeric sequences were identified and eliminated using the UCHIME (v4.2) algorithm [24]. Representative sequences from each OTU were assigned using the SILVA database (SSU138.1) with the RDP classifier (v2.1.4), with the confidence threshold set to 0.8 [25]. Alpha-diversity indices were calculated to assess the richness and evenness of the microbial communities. Principal coordinate analysis (PCoA), based on the Bray–Curtis dissimilarity matrix, Unweighted UniFrac distance, and Weighted UniFrac distance, was employed to elucidate the differences between microbial communities. The analysis of similarities (ANOSIM) and Adonis, implemented in Vegan (v2.6-8) [26], were used to evaluate group similarity and the strength and significance of microbial communities. Tax4Fun (v0.3.1) was employed to predict potential microbial functions, which were categorized based on the Kyoto Encyclopedia of Genes and Genomes (KEGG) pathways [27].

### 2.3. Measurement of Fecal Metabolites and Data Analysis

Fecal metabolites (25 mg per sample) were determined using previously described methods [28]. Briefly, the extraction of metabolites was conducted using 500 μL of a methanol/acetonitrile/water solution (in a ratio of 2:2:1 by volume). The supernatant was analyzed using a Vanquish ultra-high-performance liquid chromatography (UHPLC) system (Thermo Fisher Scientific, Waltham, MA, USA), interfaced with an Orbitrap Q Exactive series mass spectrometer (Thermo Fisher Scientific, Waltham, MA, USA). For the UHPLC system, a binary mobile phase was employed: phase A comprised 25 mmol/L ammonium acetate and 25 mmol/L ammonia hydroxide, while phase B was acetonitrile. Chromatographic separation was executed on a Waters ACQUITY UPLC BEH Amide column (2.1 mm × 100 mm, 1.7 μm) at 45 °C and a flow rate of 0.3 mL/min. Mass spectrometry analysis was performed under the following conditions: a sheath gas flow rate of 50 Arb, an auxiliary gas flow rate of 15 Arb, a capillary temperature of 350 °C, a full MS resolution of 60,000, an MS/MS resolution of 15,000, collision energy settings of 20/30/40 in NCE mode, and a spray voltage of 3.8 kV for positive mode, or −3.4 kV for negative mode.

The raw UHPLC-MS/MS data were converted to mzXML format using ProteoWizard (v3) [29]. Subsequent steps, including peak identification, extraction, alignment, and integration, were performed using XCMS (v4.4.0) [30], based on the KEGG and HDMB datasets. Principal component analysis (PCA) and partial least squares discriminant analysis (PLS-DA) were utilized to reveal differences in fecal metabolites. Metabolites with significant differences were identified based on variable importance in the projection (VIP > 1.0) and *p*-values of <0.05, as determined by the Kruskal–Wallis test. A KEGG enrichment analysis of significantly altered metabolites was performed using MetaboAnalyst 5.0 [31].

### 2.4. Statistical Analysis

The differences in microbial diversity indices, relative abundances in the microbial composition, KEGG pathways, and concentrations of metabolites between the Cap and Gra groups were determined by performing the Kruskal–Wallis test. Significance was corrected using the Benjamini–Hochberg correction for multiple comparisons. The data are presented as the mean ± standard error of the mean (SEM). To explore the relationships between the significantly different microbiota and metabolites in the fecal samples, the Spearman correlation coefficient was calculated using Hmisc (v5.1-3) [32]. Correlations were considered significant if the absolute value of the rho coefficient was >0.8 and the *p*-value was ≤0.05, in which case they were then visualized using Gephi (v0.9.6) [33].

## 3. Results

### 3.1. The Microbial Diversity and Composition of Feces in the Cap and Gra Groups

**Figure 1:**
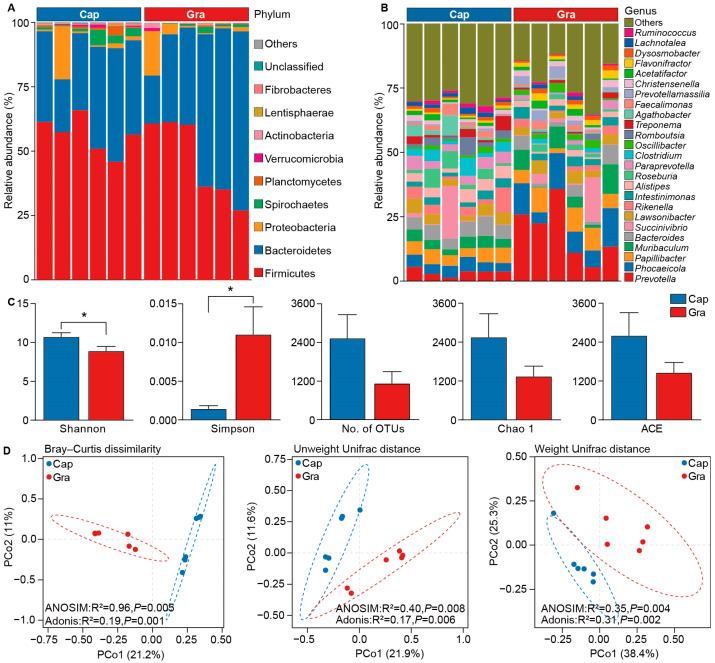
Microbial community composition and diversity in the feces of captive and grazing reindeer. Microbial community composition in the feces of the Cap and Gra groups at the phylum (**A**) and genus (**B**) levels. (**C**) A comparison of alpha-diversity indices in feces between the Cap and Gra groups. (**D**) PCoA illustrating the differences in microbial community membership and structure in reindeer feces between the Cap and Gra groups at the OTU level, based on Bray–Curtis dissimilarity, Unweighted UniFrac distance, and Weighted UniFrac distance. * *p* < 0.05.

This study generated a total of 61,158 and 63,014 full-length 16S rRNA gene sequences, with an average of 10,193 and 10,502 sequences for the Cap and Gra groups, respectively. Based on a 98.65% sequence similarity, a total of 19,504 OTUs were identified, comprising 14,400 and 5185 OTUs from the Cap and Gra groups, respectively. Firmicutes (Cap = 56.32 ± 2.92%, Gra = 46.93 ± 6.40%), Bacteroidetes (Cap = 34.32 ± 3.36%, Gra = 46.99 ± 8.08%), and Proteobacteria (Cap = 4.30 ± 3.27%, Gra = 4.11 ± 2.69%) were the predominant phyla in the feces (Figure 1A). The dominant genera in the Cap group were *Bacteroides* (5.63 ± 0.47%), *Papillibacter* (5.01 ± 0.43%), *Phocaeicola* (4.28 ± 0.36%), *Rikenella* (4.04 ± 1.24%), and *Lawsonibacter* (3.93 ± 0.59%), collectively accounting for 22.89% of the total microbial abundance (Figure 1B). In the Gra group, the dominant genera were *Prevotella* (18.97 ± 4.56%), *Phocaeicola* (10.02 ± 1.76%), *Papillibacter* (6.69 ± 1.26%), *Muribaculum* (5.59 ± 1.81%), and *Succinivibrio* (3.84 ± 2.73%), constituting 45.11% of the total microbial abundance.

The Shannon index in the Cap group was significantly higher (*p* < 0.05, H = 4.33) than that in the Gra group, while the Simpson index was significantly lower (*p* < 0.05, H = 5.06). However, the differences in the number of OTUs and the Chao 1 and ACE indices between the Cap and Gra groups were not significant (*p* > 0.05, Figure 1C). PCoA based on the Bray–Curtis dissimilarity matrix (ANOSIM, *p* = 0.005; Adonis, *p* = 0.001), Unweighted UniFrac distance (ANOSIM, *p* = 0.008; Adonis, *p* = 0.006), and Weighted UniFrac distance (ANOSIM, *p* = 0.004; Adonis, *p* = 0.002) revealed that the microbial community membership and structure were significantly different between the Gra and Cap groups (Figure 1D).

### 3.2. Differences in Fecal Microbial Composition and Functions Between the Cap and Gra Groups

**Figure 2:**
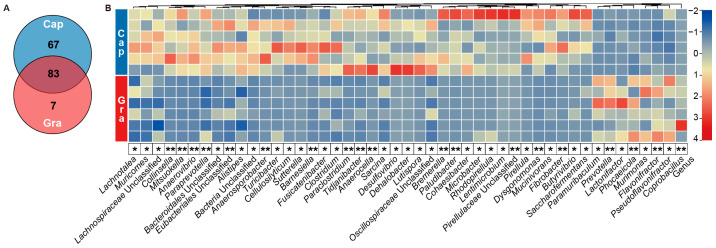
The significantly different genera in the feces of captive and grazing reindeer. (**A**) A Venn diagram illustrating genera that were common and unique to the Cap and Gra groups. (**B**) A heatmap depicting the significantly different genera in feces between the Cap and Gra groups. Individuals are shaded from blue to red to represent relative abundances (low to high). * *p* < 0.05 and ** *p* < 0.01.

A total of 83 genera were common to both groups, while 67 and 7 genera were unique to the Cap or Gra group, respectively (Figure 2A). Furthermore, the relative abundances of 47 genera significantly differed between the two groups (*p* < 0.05, H > 3.86). The Cap group had significantly higher relative abundances of *Clostridium* (Cap = 3.49 ± 0.86%, Gra = 0.71 ± 0.36%), *Paraprevotella* (Cap = 3.89 ± 0.43%, Gra = 1.34 ± 0.48%), *Alistipes* (Cap = 3.79 ± 0.54%, Gra = 1.78 ± 0.36%), *Paludibacter* (Cap = 1.60 ± 0.55%, Gra = 0.01 ± 0.01%), *Mitsuokella* (Cap = 0.56 ± 0.11%, Gra = 0.0), *Lentimicrobium* (Cap = 0.50 ± 0.36%, Gra = 0.0), *Paraclostridium* (Cap = 0.41 ± 0.12%, Gra = 0.02 ± 0.01%), and *Anaerovibrio* (Cap = 0.29 ± 0.05%, Gra = 0.0) compared to the Gra group (*p* < 0.05, H > 3.86). However, the relative abundances of *Prevotella* (Cap = 3.46 ± 0.56%, Gra = 18.97 ± 4.56%), *Phocaeicola* (Cap = 4.28 ± 0.36%, Gra = 10.02 ± 1.76%), *Flavonifractor* (Cap = 0.75 ± 0.10%, Gra = 2.05 ± 0.31%), *Pseudoflavonifractor* (Cap = 0.56 ± 0.13%, Gra = 1.33 ± 0.26%), *Paramuribaculum* (Cap = 0.0, Gra = 0.09 ± 0.03%), *Coprobacillu* (Cap = 0.0, Gra = 0.12 ± 0.07%), *Murimonas* (Cap = 0.0, Gra = 0.05 ± 0.02%), and *Lactonifactor* (Cap = 0.0, Gra = 0.13 ± 0.05%) were significantly higher in the Gra group than in the Cap group (*p* < 0.05, H > 3.86, Figure 2B).

**Figure 3:**
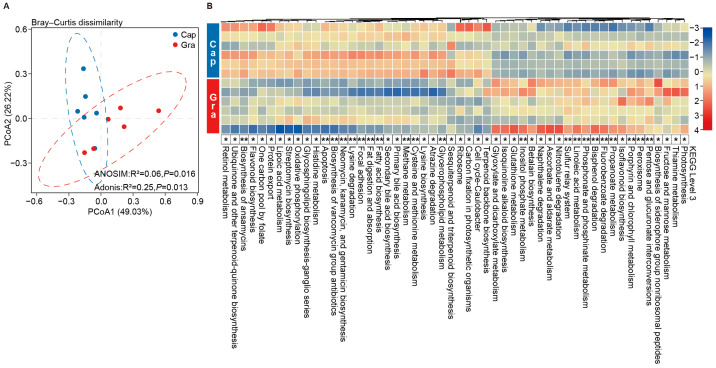
A comparison of the potential functions of microbes in the feces of captive and grazing reindeer. (**A**) PCoA illustrating the variation in microbial functions at KEGG level 3, based on the Bray–Curtis dissimilarity matrix, in feces between the Cap and Gra groups. (**B**) A heatmap showing the significantly different metabolic pathways of fecal microbiota between the Cap and Gra groups. Individuals are shaded from blue to red to indicate relative abundances (low to high). * *p* < 0.05 and ** *p* < 0.01.

The PCoA results of KEGG level 3, based on the Bray–Curtis dissimilarity matrix, indicate that the functional profiles significantly differed between the Cap and Gra groups (ANOSIM, *p* = 0.016; Adonis, *p* = 0.013, Figure 3A), with a total of 52 significantly different pathways (*p* < 0.05, H > 4.33). The relative abundances of glycerophospholipid metabolism, fatty acid biosynthesis, fat digestion and absorption, primary bile acid biosynthesis, secondary bile acid biosynthesis, histidine biosynthesis, lysine biosynthesis, lysine degradation, and cysteine and methionine metabolism pathways were significantly higher in the Cap group compared to the Gra group (*p* < 0.05, H > 4.33). However, the relative abundances of fructose and mannose metabolism, pentose and glucuronate interconversion, propanoate metabolism, inositol phosphate metabolism, glyoxylate and dicarboxylate metabolism, and ascorbate and aldarate metabolism pathways were greater in the Gra group (*p* < 0.05, H > 4.33, Figure 3B).

### 3.3. Variations in Fecal Metabolites Between the Cap and Gra Groups

**Figure 4:**
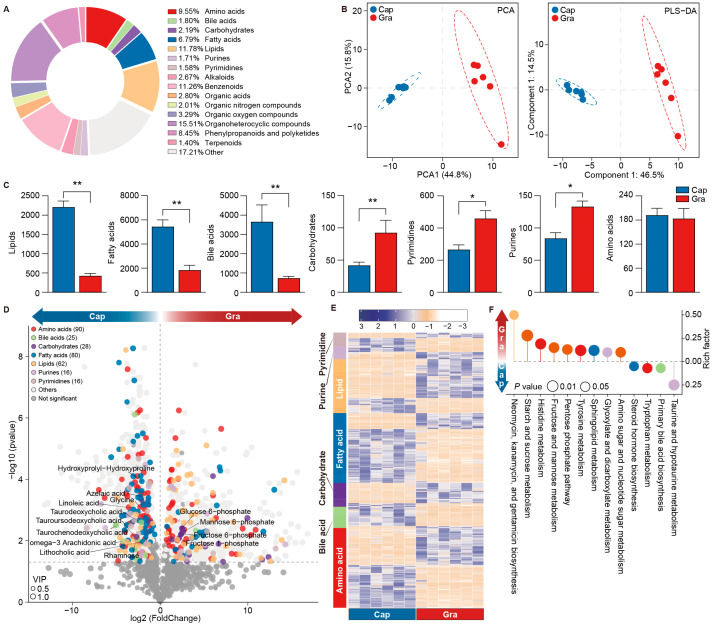
Differences in fecal metabolites between captive and grazing reindeer. (**A**) A pie chart illustrating the classification of identified metabolites in feces. (**B**) PCA and PLS-DA plots highlighting the differences in fecal metabolites between the Cap (blue) and Gra (red) groups. (**C**) A comparison of the total concentrations of lipids, fatty acids, bile acids, carbohydrates, purines, pyrimidines, and amino acids between the Cap and Gra groups. (**D**) Volcano plots depicting the significantly different metabolites in feces between the Cap and Gra groups. (**E**) A heatmap showing the significantly different metabolites in reindeer feces when comparing the Gra group to the Cap group. Individuals are shaded from yellow to purple to indicate concentrations (low to high). (**F**) A lollipop chart displaying the enriched metabolic pathways of significantly different metabolites. * *p* < 0.05 and ** *p* < 0.01.

We subsequently characterized the fecal metabolites in the Cap and Gra groups through UHPLC-MS/MS analysis, and identified a total of 2283 metabolites, including 1347 and 936 metabolites in the positive and negative ion modes, respectively. These metabolites were classified into amino acids, bile acids, carbohydrates, fatty acids, lipids, purines, pyrimidines, alkaloids, benzenoids, and organic acids (Figure 4A, Table S2). The PCA and PLS-DA showed a clear separation of fecal metabolites between the Cap and Gra groups (Figure 4B). The total concentration of lipids, fatty acids, and bile acids in the feces of the Cap group was higher than that of the Gra group, while the concentration of carbohydrates, purines, and pyrimidines was lower in the former group (*p* < 0.05, H > 6.56, Figure 4C). A total of 1269 metabolites were significantly different, with 624 and 645 metabolites increased in the Cap and Gra groups, respectively (*p* < 0.05, H > 4.33). The significantly different metabolites included 90 amino acids, 25 bile acids, 28 carbohydrates, 80 fatty acids, 62 lipids, 16 purines, and 16 pyrimidines (Figure 4D, Table S3). Additionally, the concentrations of O-succinyl-L-homoserine, glycine, rhamnose, isodeoxycholic acid, lithocholic acid, chenodeoxycholic acid, taurochenodeoxycholic acid, (Z)-6-octadecenoic acid, linoleic acid, myristic acid, and azelaic acid in the feces of the Cap group were significantly increased relative to the Gra group, whereas the concentrations of glutamate, valine, D-ribose, palmitamide, and dilinolenin (9c,12c,15c) were significantly decreased (Figure 4E, Table S4). The significantly increased metabolites in the Cap group were enriched in primary bile acid biosynthesis and steroid hormone biosynthesis, whereas the increased metabolites in the Gra group were enriched in starch and sucrose metabolism, fructose and mannose metabolism, the pentose phosphate pathway, amino sugar and nucleotide sugar metabolism, sphingolipid metabolism, histidine metabolism, and tyrosine metabolism (*p* < 0.05, Figure 4F).

### 3.4. Co-Occurrence of Fecal Microbiota and Metabolites in the Cap and Gra Groups

**Figure 5:**
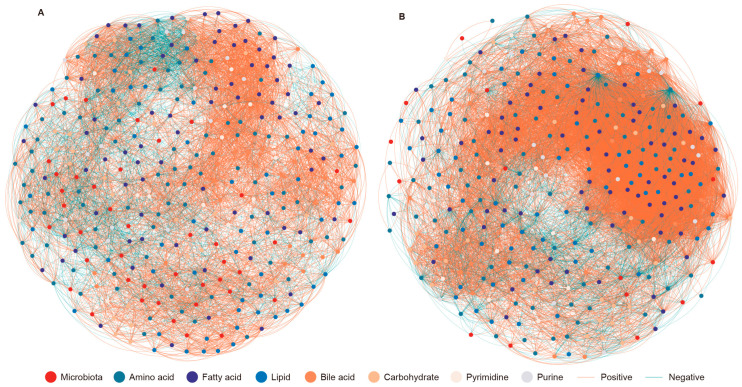
The co-occurrence of significantly different microbiota and metabolites in the feces of captive (**A**) and grazing (**B**) reindeer. The Spearman correlation coefficient (|rho| > 0.8 and *p* ≤ 0.05) was calculated from the abundances of microbiota and the concentrations of metabolites. Node colors indicate microbiota and metabolites, with yellow and blue edges representing positive and negative correlations, respectively.

We investigated the correlations between the significantly altered microbiota and metabolites in the Cap and Gra groups, respectively (Figure 5). The co-occurrence network of the Cap group comprised 354 nodes and 7362 edges, whereas that of the Gra group included 326 nodes and 15,276 edges. A comparison of the network parameters indicated that the eigenvector centrality and average path length were higher in the Cap group, while the average degree, average weighted degree, average clustering coefficient, and density were lower compared to the Gra group (Table S5). In the Cap group, the genera *Clostridium*, *Paraprevotella*, and *Paludibacter* were positively correlated with D-ribose. Lithocholic acid exhibited positive correlations with O-succinyl-L-homoserine, isodeoxycholic acid, chenodeoxycholic acid, and *Anaerosporobacter* (Table S6). In the Gra group, *Prevotella* and *Phocaeicola* were positively correlated with *Paramuribaculum* and negatively correlated with lithocholic acid, taurochenodeoxycholic acid, and *Clostridium*. *Alistipes* was negatively correlated with D-ribose, isodeoxycholic acid, chenodeoxycholic acid, and *Clostridium* (Table S7).

## 4. Discussion

In this study, we investigated differences in the fecal microbiota and metabolites between captive and grazing male reindeer using full-length 16S rRNA gene sequencing and UHPLC-MS/MS. The phyla Firmicutes, Bacteroidetes, and Proteobacteria were predominant in the feces of the reindeer, which is consistent with findings on the fecal microbiota of adult grazing male and female Svalbard reindeer during summer [12], suggesting similarities in the gastrointestinal tract microbiota at the phylum level in adult male reindeer across different regions. Our results show that the genera *Bacteroides* and *Prevotella* were dominant in the feces of captive and grazing male reindeer, respectively, while the genera *Papillibacter* and *Phocaeicola* were common to both groups. This is partially consistent with the findings of Zielińska et al. (2016), who found that *Bacteroides* was dominant in the feces of adult grazing male reindeer during the summer in the Hornsund fjord [12]. A correlation between spatial location and fecal microbiota composition was observed in white-tailed deer [34]. These results indicate the potential effects of regional distribution on the composition of gut microbiota at the lower taxonomic levels. The difference between the results of this study and the previous findings is likely related to the sequencing approaches and taxonomic classification, because full-length 16S rRNA gene sequencing can enhance taxonomic resolution [35], as well as the limited sample size of animals used in this study. Phylogenetic analysis has demonstrated that *B. vulgatus*, *B. dorei*, and *B. massiliensis* are closely related to *Phocaeicola*, and they have been reclassified as *P. vulgatus*, *P. dorei*, and *P. massiliensis* [36]. However, it is well known that diet plays a significant role in shaping the gut microbiota [37]. Mönttinen et al. (2022) also found significant differences in microbial diversity and composition in feces between captive and grazing female reindeer during winter [22]. Increased access to different types of food by free-living populations results in greater microbial diversity, while the digestion of concentrated diets by captive populations affects and shapes the gut microbiota [15,38,39]. A higher proportion of *Bacteroides* was observed in captive populations of white-tailed deer, which was related to the influence of the supplied diet and rearing conditions [40]. *Bacteroides* exhibits remarkable substrate flexibility, capable of degrading dietary or host-derived glycans and utilizing dietary amino acids as energy sources [41]. *Papillibacter*, a butyrate-producing bacterium, is associated with enhanced feed utilization efficiency [42], and *Phocaeicola* is able to digest xylan, a component widely present in plant cell walls [43]. These findings indicate that the fecal microbiota is closely linked to carbohydrate and amino acid metabolism in captive and grazing male reindeer during summer. It has been reported that the digestibility and adipocyte volume of reindeer peak during summer [44,45]. Interestingly, the gut microbiota is an important factor that affects energy harvested from the diet and fat storage in the host [13]. These results suggest that the gut microbiota likely contributes to energy storage and fat reserves in male reindeer during summer.

The microbial diversity indices of the feces of captive reindeer were significantly higher than those of grazing reindeer. Mönttinen et al. (2022) reported similar results, that the Shannon index of fecal microbiota in captive reindeer during winter was higher than in grazing reindeer [22]. Moreover, the microbial diversity in the feces of captive red deer, sika deer, white-lipped deer, and Père David’s deer was also significantly higher than that in wild populations [17,20,38,46]. However, the microbial composition in the gastrointestinal tract was not significantly different between semi-domesticated and wild Svalbard reindeer [47]. Shabat et al. (2016) demonstrated that a lower richness in rumen microbial gene content and taxa in cows was closely associated with higher feed efficiency, leading to improved energy and carbon utilization [48]. These findings imply that grazing reindeer have increased fermentation capacity and efficiency relative to captive reindeer.

The relative abundances of *Clostridium*, *Paraprevotella*, *Alistipes*, *Paludibacter*, *Mitsuokella*, *Lentimicrobium*, *Paraclostridium*, and *Anaerovibrio* were higher in the feces of captive male reindeer. Li et al. (2024) found that *C. sporogenes* in the mouse gut encodes two arginine deiminase genes and four cysteine desulfurase genes [49]. Fonknechten et al. (2010) demonstrated that *C. stricklandii* preferentially utilizes threonine, arginine, and serine for energy production [50]. Moreover, it was reported that *C. scindens* can metabolize primary bile acids into secondary bile acids through 7α-dehydroxylation [51]. These results suggest the key roles of *Clostridium* in amino acid utilization in the gut of captive male reindeer. Radka et al. (2020) showed that *Alistipes finegoldii* utilizes medium- and long-chain fatty acids to assemble membrane lipids in the human gut [52]. A recent study showed that *Paraclostridium* was significantly increased in the ilea of dairy cows fed a high-grain diet [53]. It is known that *Anaerovibrio* encodes three lipase genes (*alipA*, *alipB*, and *alipC*) that hydrolyze lipids [54], and increased abundances of *Paludibacter* and *Clostridium* were found in the rumen of cattle fed a high-grain diet [55,56]. Consistently, the predicted microbial functions showed that the pathways related to histidine metabolism, lysine biosynthesis, cysteine and methionine metabolism, glycerophospholipid metabolism, fatty acid biosynthesis, fat digestion and absorption, primary bile acid biosynthesis, and secondary bile acid biosynthesis were enriched in the feces of captive reindeer. Additionally, the total concentrations of lipids, amino acids, fatty acids, and bile acids were increased in the feces of captive reindeer. The supplementation of high-grain diets resulted in elevated levels of amino acids, such as leucine, glycine, and alanine [57]. Bile acids, including primary and secondary bile acids, facilitate lipid digestion [58]. These findings suggest that the utilization of amino acids and fatty acids is likely enhanced in the gut of captive male reindeer.

In the feces of the grazing reindeer, the relative abundances of *Prevotella*, *Phocaeicola*, *Flavonifractor*, *Pseudoflavonifractor*, *Paramuribaculum*, *Coprobacillu*, *Murimonas*, and *Lactonifactor* were increased. *Prevotella* is a diverse genus in the mammalian gut, encoding several genes for the degradation of complex carbohydrates, such as acetylxylan esterase, pectate lyase, alpha-L-fucosidase, 1,4 beta-xylanase, and phosphoenolpyruvate carboxykinase [59]. Previous studies have revealed that the increased abundance of *Flavonifractor* enhances microbial glycolysis and polysaccharide degradation [60]. *Flavonifractor plautii* and *Paramuribaculum* are known to produce propionate and butyrate as fermentation products [61,62], and *Phocaeicola vulgatus* has been identified as a potential propionate producer [63]. *Murimonas* is an acetate-producing bacterium that utilizes substrates such as D-fructose, D-galacturonic acid, D-malic acid, L-alanyl-L-threonine, and L-glutamic acid [64]. Lesniak et al. (2022) demonstrated that *Lactonifactor* is actively involved in fiber degradation in mouse feces [65]. Moreover, the abundance of *Pseudoflavonifractor* was significantly increased in the ceca of yaks when their diets were supplemented with alfalfa hay [66]. In this study, the relative abundances of fructose and mannose metabolism, pentose and glucuronate interconversion, and propanoate metabolism pathways were significantly higher in grazing reindeer than in captive reindeer. Consistently, the rumen microbiota of yaks living on the Qinghai–Tibet Plateau was enriched in the pentose and glucuronate interconversion pathway [67]. These results suggest the enhanced ability of grazing reindeer to utilize plant fibers.

Our study shows that the microbial populations are distinct between male captive and grazing reindeer, which may be useful for reindeer breeders, as the gut microbiota is important for the health and productivity of livestock [68], and could be leveraged to improve spermatogenesis and sperm motility in order to treat male infertility [69]. However, it is noted that the findings were obtained from male reindeer with a limited sample size, indicating the importance and necessity of completing more investigations comparing the gut microbial composition and metabolic functions between male and female reindeer, and between summer and winter, based on a large sample size. Wildlife plays an important role in maintaining ecological balance and biodiversity; however, this is challenged by the varying availability and nutritional content of feed associated with changes in climate and geography [15,70]. Thus, the culture and application of significantly enriched gut microorganisms could improve nutrient utilization efficiency and ensure the conservation of reindeer.

## 5. Conclusions

In this study, we identified significant differences in fecal microbial communities and metabolites between captive and grazing male reindeer during summer, indicating the impact of captivity on gut microbiota. The fecal microbiota of captive male reindeer was associated with amino acid and fatty acid utilization, while the utilization of fiber plant materials was enhanced in the fecal microbiota of grazing male reindeer. This study highlights the influence of dietary components and management on gut microbiota. These findings are likely to provide new insights into feeding strategies and management practices, in order to optimize growth performance and support the sustainable conservation of reindeer populations.

## Data Availability

Raw sequence reads for all samples are available under NCBI project PRJNA1152284.

## Supplementary Materials

The following supporting information can be downloaded at https://www.mdpi.com/article/10.3390/ani14243606/s1. Table S1: Ingredients and nutrient composition of the diet (%, DM basis); Table S2: The metabolites in positive and negative ion modes; Table S3: The classification of significantly different metabolites; Table S4: The significantly different metabolites in grazing reindeer vs. captive reindeer; Table S5: The co-occurrence network parameter of captive and grazing reindeer; Table S6: The correlations of significantly different microbiota and metabolites in the feces of captive reindeer; Table S7: The correlations of significantly different microbiota and metabolites in the feces of grazing reindeer.
